# Real-Time Pupil Localization Algorithm for Blurred Images Based on Double Constraints

**DOI:** 10.3390/s25061749

**Published:** 2025-03-12

**Authors:** Shufang Qiu, Yi Wang, Zeyuan Liu, Huaiyu Cai, Xiaodong Chen

**Affiliations:** Key Laboratory of Opto-Electronics Information Technology of Ministry of Education, School of Precision Instruments and Optoelectronic Engineering, Tianjin University, Tianjin 300072, China; 3018202321@tju.edu.cn (S.Q.); 1023202012@tju.edu.cn (Z.L.); hycai@tju.edu.cn (H.C.); xdchen@tju.edu.cn (X.C.)

**Keywords:** eye tracker, blurred images, pupil center localization, grayscale constraints, geometric constraints, pupil shape index

## Abstract

Accurate pupil localization is crucial for the eye-tracking technology used in monitoring driver fatigue. However, factors such as poor road conditions may result in blurred eye images being captured by eye-tracking devices, affecting the accuracy of pupil localization. To address the above problems, we propose a real-time pupil localization algorithm for blurred images based on double constraints. The algorithm is divided into three stages: extracting the rough pupil area based on grayscale constraints, refining the pupil region based on geometric constraints, and determining the pupil center according to geometric moments. First, the rough pupil area is adaptively extracted from the input image based on grayscale constraints. Then, the designed pupil shape index is used to refine the pupil area based on geometric constraints. Finally, the geometric moments are calculated to quickly locate the pupil center. The experimental results demonstrate that the algorithm exhibits superior localization performance in both blurred and clear images, with a localization error within 6 pixels, an accuracy exceeding 97%, and real-time performance of up to 85 fps. The proposed algorithm provides an efficient and precise solution for pupil localization, demonstrating practical applicability in the monitoring of real-world driver fatigue.

## 1. Introduction

In recent years, road traffic safety has become an increasingly serious issue. More than 1.3 million people die in road traffic accidents annually, and more than 20 million people suffer non-fatal injuries as a result of these accidents [1]. Statistics show that fatigue driving is one of the leading causes of traffic accidents [2]. Consequently, the effective monitoring of driver fatigue has become critical for improving road safety and reducing traffic-related fatalities. With the rapid development of computer vision technology, eye-tracking-based driver fatigue monitoring systems [3] have gradually become a research hotspot. Eye trackers determine the center of the pupil to calculate indicators such as the driver’s gaze point and eye movement speed, enabling the real-time monitoring of the driver’s condition. However, factors such as poor road conditions can cause vehicle vibrations, resulting in blurred images being captured by the eye tracker [4]. In addition, the field of view of the image captured by the eye tracker is large, with the eye region occupying only a small portion of the image pixels, and the resolution is relatively low [5]. These factors may cause the eye tracker to capture blurred images of the human eye, which affects the precise localization of the pupil center and, consequently, reduces the reliability of driver fatigue monitoring. Therefore, developing real-time pupil localization algorithms for blurred images is of great value in monitoring driver fatigue.

Pupil localization algorithms can be classified into two categories: data-driven methods and knowledge-based methods [6,7]. Data-driven methods learn the inherent patterns and features of data through extensive training on large datasets, thereby accomplishing the task of pupil localization [8]. These methods demonstrate superior performance when processing complex scenes and blurred images. For instance, a robust pupil localization algorithm based on a modified dense fully connected network has been proposed, enabling the rapid and accurate detection of the pupil center even in the presence of reflections and occlusions [9]. Additionally, a Pupil-Locator Network (PLNet), which utilizes domain-specific data augmentation, has been developed to address challenging conditions such as reflections, exposure variations, and blur, further enhancing the robustness of pupil localization [10]. However, data-driven methods rely on the support of datasets [11], and the existing datasets struggle to fully cover human eye images with varying levels of blur, resulting in limited model generalization. In addition, the model inference and data processing steps require more computational resources, which may affect the response times of driver fatigue monitoring systems when hardware resources are constrained.

Traditional knowledge-based methods perform pupil localization based on prior knowledge, such as low grayscale values, circular shapes, and a lack of texture within the pupil. These methods can be categorized into three types based on the features utilized by the algorithms: grayscale-based [12,13], gradient-based [14,15,16], and shape-based methods [17,18]. A grayscale-based approach using the Gray Projecting Integral Method (GPIM) [13] for pupil localization offers flexibility but suffers from reduced accuracy under poor lighting and in complex scenes. To improve localization precision, a gradient-based approach utilizing the Hough Transform (HT) [14] detects the pupil center with high accuracy and robustness against interference. However, its one-to-many mapping results in high computational complexity and poor real-time performance. In contrast, shape-based approaches, such as the Fast Radial Symmetry Transform (FRST) [18], enhance real-time efficiency by reducing algorithmic complexity through parameter space conversion. These algorithms achieve accurate pupil localization in clear eye images.

However, traditional methods designed for clear images face significant challenges when applied to blurred images of human eyes. Although the overall grayscale distribution trend and large-scale shape information are preserved, high-frequency components such as texture features and edge features undergo significant losses, and local grayscale detail features and small-scale shape features are weakened. As a result, conventional pupil localization algorithms are ineffective in processing blurred images. To address this, Zheng et al. [19] proposed a human eye location method based on blurred infrared images. First, the image is enhanced by adaptive histogram equalization to compensate for the loss of gray information, and then the human eye region is located through template matching. While this method yields good localization results for blurred images, it targets the human eye region rather than the pupil center. Li et al. [20] proposed a coarse-to-fine positioning algorithm that combines grayscale, gradient, and other information. The algorithm consists of four steps: coarse positioning using the Otsu method, edge positioning using gradient features, edge repositioning using sub-pixel methods, and ellipse fitting. The algorithm effectively improves the pupil localization accuracy of blurred images, but it is time-consuming and does not meet the real-time requirements for monitoring driver fatigue.

To achieve accurate pupil localization in blurred images while ensuring real-time performance, this paper proposes a pupil localization algorithm based on double constraints. First, the rough pupil area is extracted from the whole image based on grayscale constraints to reduce the interference of invalid information. Subsequently, a specifically designed pupil shape index serves as the key condition for screening the pupil area, and the pupil area is refined based on geometric constraints. This approach avoids the complex computations involved in edge fitting and mitigates the loss of high-frequency components caused by image blur. Finally, the geometric moments of the region are directly used to locate the pupil center, reducing computational time. The experimental results demonstrate that the proposed algorithm achieves precise pupil localization in blurred human eye images, improving both the localization speed and accuracy, thereby enhancing the technology used for real-time driver fatigue monitoring.

## 2. Proposed Method

Near-infrared imaging is often used for on-board monitoring [21] because near-infrared imaging can stably capture pupil features under various lighting conditions, especially in accident-prone nighttime environments. In addition, the eye has a higher reflection of near-infrared light than visible light. Therefore, the pupil location algorithm proposed in this study is targeted at infrared images.

**Figure 1:**
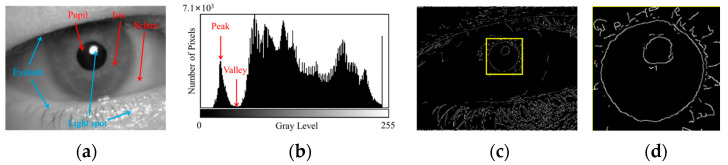
Analysis of clear human eye image features. (**a**) Periocular image, where the pupil is the region with the lowest gray levels. (**b**) Grayscale histogram, showing the distribution of pixel counts across different gray levels. The pixels corresponding to the pupil area are typically found in the first peak of the histogram, representing the lowest gray levels. (**c**) Edge detection results, and the pupil area is outlined with a yellow box. (**d**) pupillary boundary, which is not a perfect circle.

**Figure 2:**
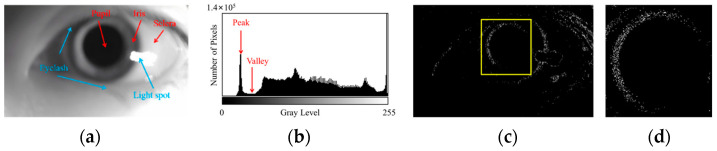
Analysis of blurred human eye image features. (**a**) Periocular image, where the texture information is weakened. (**b**) Grayscale histogram, where the overall statistical characteristics of the histogram are preserved despite blurring. (**c**) Edge detection results, and the pupil area is outlined with a yellow box. (**d**) pupillary boundary, where the edge gradient is reduced and the edge contour becomes diffuse.

The features of a clear human eye image are shown in Figure 1, and the features of a blurred human eye image are shown in Figure 2. As shown in Figure 1 and Figure 2, the pupil localization algorithm for blurred images requires the pupil area to be accurately separated from the iris and sclera areas and the pupil center to be detected in the presence of blurred edges and reduced contrast.

**Figure 3:**
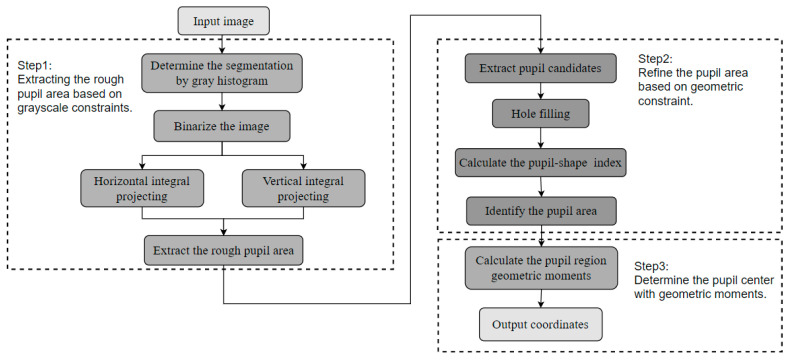
Flowchart of the proposed pupil localization algorithm.

Based on the analysis above, the flowchart of the pupil localization algorithm proposed in this article is shown in Figure 3. The algorithm consists of three steps: extracting the rough pupil area based on grayscale constraints, refining the pupil region based on geometric constraints, and determining the pupil center using geometric moments. First, the statistical information of the grayscale histogram is combined with grayscale integral projection as grayscale constraints to extract the rough pupil area. Next, connected components are extracted, and morphological hole filling is performed to mitigate the impact of reflective artifacts. The designed pupil shape index is then used as the screening condition of the connected components, and the pupil area is refined through geometric constraints. Finally, the geometric moment of the pupil area is calculated to output the coordinates of the pupil center.

### 2.1. The Extraction of the Rough Pupil Area Based on Grayscale Constraints

**Figure 4:**
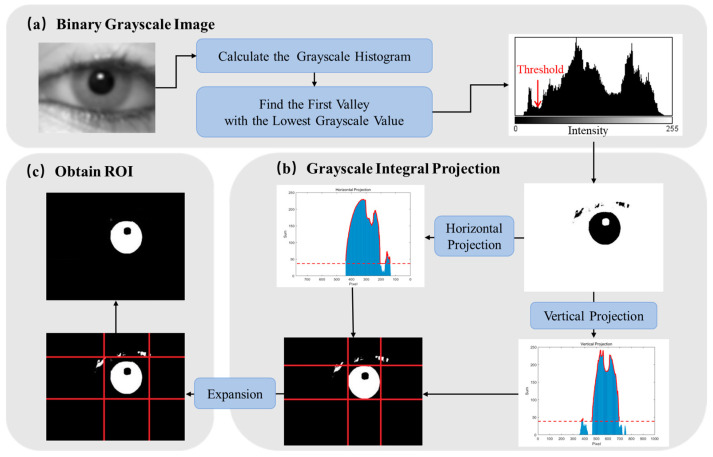
Process of extracting the rough pupil area. (**a**) The segmentation threshold is determined using the statistical information of the gray histogram, and the gray image is transformed into a binary image. (**b**) Then, a rectangular mask is obtained using the gray integral projection method. The red dashed line indicates a threshold set at 15% of the maximum value, which is used to determine the main portion of the grayscale integral projection and define the mask. (**c**) Finally, the rectangular mask is expanded outward in a particular proportion to obtain the rough pupil area.

Due to factors such as uneven illumination, dark areas and reflected spots appear in the image. Additionally, interference from hair and eyebrows may also be present. Therefore, it is necessary to find the rough pupil region from the whole image. Due to the high complexity of the scene in the image to be processed, a fixed-position and fixed-size region mask cannot provide satisfactory results. Therefore, we propose a method to extract the rough pupil region based on grayscale constraints, as shown in Figure 4. This step relies solely on grayscale statistical information, thereby minimizing interference caused by image blurring. Moreover, only one traversal of the image pixels is required, allowing for efficient use of the grayscale information and reducing computational complexity.

**Figure 5:**
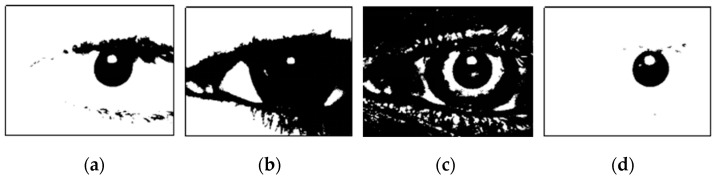
Results of different threshold segmentation methods. (**a**) The empirical threshold method. Here, the segmentation thresholds are fixed, making this method less robust. (**b**) The maximum inter-class variance method. This method assumes that all pixels are the two most different types, but it cannot ensure that the pupil region is just one of them. (**c**) The local mean method. This method calculates the mean value in the local neighborhood of each pixel, which is computationally intensive and produces unnecessary over-segmentation. (**d**) The histogram statistical method.

Firstly, the segmentation threshold is determined using the statistical information from the gray histogram. Based on prior knowledge, the pixel values of the pupil area are low, and the pupil area occupies a specific region. Regardless of the ambient lighting conditions or image clarity, the pixels of the pupil region are generally contained in the spikes with low gray values. Therefore, we set the segmentation threshold at the gray value corresponding to the valley of the lowest peak in the histogram. The binarization results generated using different threshold segmentation methods are shown in Figure 5. The result of the threshold segmentation using the histogram statistical method (Figure 5d) generally includes the pupil area and dark areas with similar grayscale values. This method adapts to the pupil region’s gray range based on statistical information, making it both simple and effective.

After obtaining the binarization result using the histogram statistical method (Figure 5d), the image is subjected to grayscale integral projection along both the vertical and horizontal directions. By traversing and accumulating the number of pixels with zero grayscale value in these directions, two one-dimensional grayscale cumulative distribution curves are generated. The coordinate values corresponding to the main regions of the two curves help to determine the rough pupil region in both dimensions, resulting in the creation of a rectangular mask. To account for potential interference, such as reflected light spots near the pupil boundary, which may lead to an undersized rough pupil area, the rectangular mask is expanded by 1/20 of the number of pixels along the long side of the image.

The method of extracting the rough pupil area based on grayscale constraints leverages the statistical characteristics of gray information in the human eye image, and the threshold does not need to be manually selected. It is both robust and adaptable, effectively reducing the impact of image blur on pupil localization. Furthermore, the calculations of the grayscale histogram and the grayscale integral projection can be performed simultaneously during the pixel traversal process, allowing for significant improvements in the algorithm’s real-time performance through parallelization, especially for large-scale human eye images.

### 2.2. Refinement of the Pupil Area Based on Geometric Constraints

**Figure 6:**
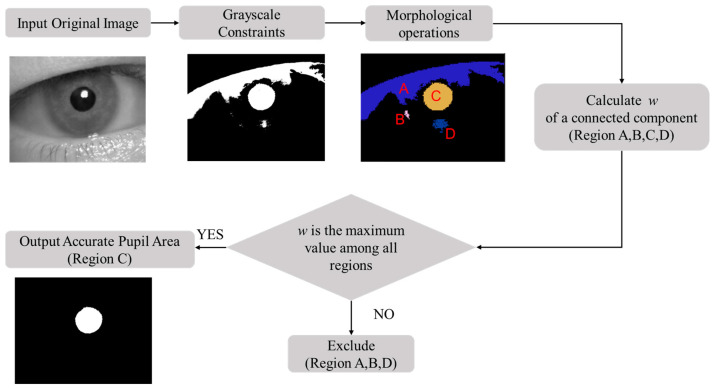
Process of refining the pupil area based on geometric constraints.

The rough pupil region extracted using gray constraints does not meet the accuracy requirements of pupil localization in monitoring driver fatigue, so further refinement of the pupil area is needed. In this study, we use morphological operations and the pupil shape index as geometric constraints to refine the pupil area, and the process is shown in Figure 6. First, the rough pupil area is preprocessed via a morphological operation. The connected regions are extracted and a hole-filling algorithm is applied to eliminate the influence of reflected light spots. Then, the connected components are screened according to the proposed pupil shape index, with the region exhibiting the largest pupil shape index selected for further processing.

First, the connected components are extracted from the rough pupil area. The shape of the connected components depends on the chosen connectivity criterion. There are two types of connectivity: 4-connectivity and 8-connectivity. The 4-connectivity method is suitable for simple object segmentation, whereas 8-connectivity is more effective for segmenting complex objects with irregular boundaries. Therefore, 8-connectivity is preferable for extracting the pupil region. After extracting the connected components, an opening operation is applied to remove low-level noise and mitigate its impact. Subsequently, a hole-filling algorithm is used to fill holes that are not connected to the image boundary, thereby eliminating the interference of reflection spots. Finally, multiple complete connected components are obtained as pupil candidates.

Since the pupil area is not a perfect circle and has a certain degree of convexity and concavity on the edge, the use of a single geometric feature for screening connected components yields unsatisfactory results. There may be no pupil candidate that meets the requirements because the restrictions are too strict; alternatively, the wrong pupil candidate may be selected because the restrictions are insufficient. To address this issue, this paper designs a pupil shape index *w* to screen multiple connected components to determine the pupil area. The expression for the pupil shape index *w* is as follows:

(1) $$w=\lambda_{1}\frac{I_{\mathrm{minor}}}{I_{\mathrm{major}}}+\lambda_{2}\frac{1}{1+\left|\Delta_{FittingError}\right|}$$

where $I_{\mathrm{minor}}$ is the secondary moment of inertia, $I_{\mathrm{major}}$ is the main moment of inertia, and $\Delta_{FittingError}$ is the error between the connected component and the ellipse fitted to it. The pupil shape index *w* is composed of two terms, which capture the inherent shape characteristics of the pupil and are robust to noise and other disturbances. The first item in the formula, $\frac{I_{\mathrm{minor}}}{I_{\mathrm{major}}}$, represents the inertia rate of the connected component, which describes the uniformity of its shape. When the shape of the region is more homogeneous, the primary and secondary moments of inertia are closer, and the value of the first term is larger. The second item in the formula, $\frac{1}{1+\left|\Delta_{FittingError}\right|}$, represents the ellipticity of the connected component and the smoothness of its boundary. When the shape of the connected component is closer to an ellipse and its edges are smoother, the value of $\left|\Delta_{FittingError}\right|$ becomes smaller, and the value of the second term increases. In general, the closer the shape of the connected component is to a circle, the larger the value of the pupil shape index *w*.

Because the geometric shape of the pupil is evenly distributed, increasing the weight of the first term encourages *w* to prioritize regions with uniform distribution. By reducing the weight of the second term, a pupil area with slight occlusion or local noise can still receive a higher score. In this paper, $\lambda_{1}$ = 0.6, $\lambda_{2}$ = 0.4. The pupil shape index of each connected component is calculated, and the connected component with the highest index is selected as the pupil area.

**Figure 7:**
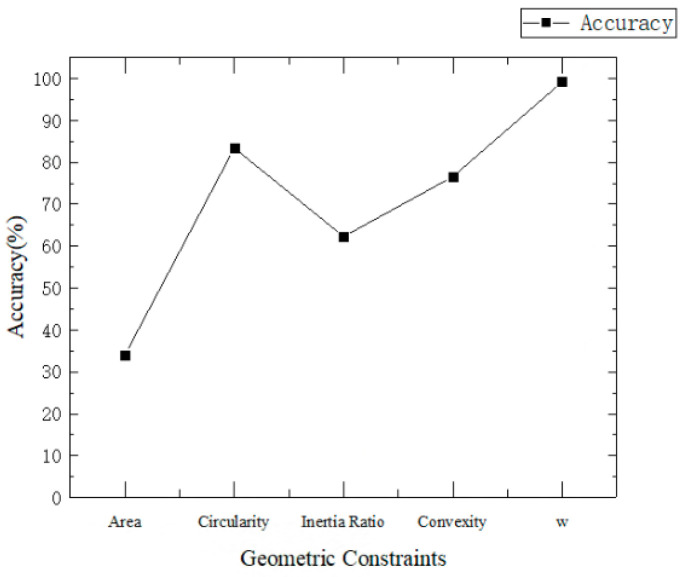
Comparison of pupil recognition accuracy across different geometric constraints.

Geometric features commonly used in image processing include the radius, area, circularity, inertia, convexity, and others. In this paper, five geometric constraints—namely, area, circularity, inertia ratio, convexity and the pupil shape index *w*—are employed to screen the pupil area. The pupil recognition accuracy using different geometric constraints is shown in Figure 7. Accuracy is defined as the ratio of the number of samples where the pupil area is correctly identified to the total number of samples. As shown in Figure 7, the accuracy achieved when using the pupil shape index *w* is higher than that obtained using any single feature.

**Figure 8:**
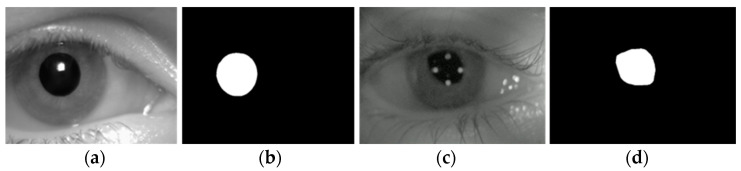
Results of pupil area refinement. (**a**) Image of regular pupils; (**b**) result of refining the regular pupil area; (**c**) image of irregular pupils; (**d**) result of refining the irregular pupil area.

The refinement of the pupil area based on geometric constraints not only enhances the accuracy of pupil recognition but also avoids the complex operation of extracting edges to calculate roundness and shape symmetry. This approach provides a solid foundation for locating the pupil center in the subsequent step. Through morphological operation, the pupil candidates become smoother and more complete. The pupil area is then screened using the proposed pupil shape index, which is robust to variations in pupil shape and aligns well with the characteristics of the pupil. The method for refining the pupil area through geometric constraints is applicable to both regular and irregular pupil regions. The results of refining the pupil area are shown in Figure 8.

### 2.3. Determination of the Pupil Center Using Geometric Moments

After obtaining the refined pupil area, the geometric moment [22] is calculated directly to determine the pupil center. The grayscale values of the binary image are treated as a two-dimensional density distribution function. The center and radius of the pupil region are then derived from the geometric moments of the connected component. The moment of a pupil area *S* can be expressed as:

(2) $$M_{\mathrm{ij}}=\sum_{x}\sum_{y}x^{i}y^{j}f(x,y)$$

where *i* and *j* are non-negative integers, (x,y)∈S, and *i* + *j* represents the order of moment *M*. *f*(*x*, *y*) represents the grayscale value of the pixel at the coordinate (*x*, *y*) in the image. The center and size of the pupil are determined by calculating the zero-order moment and the first-order moment. Specifically, the pupil center is calculated as the ratio of the first-order moment to the zero-order moment.

(3) $$x_{c}=\frac{M_{10}}{M_{00}}=\frac{\sum_{x}\sum_{y}x\;\cdot f(x,y)}{\sum_{x}\sum_{y}f(x,y)}$$

(4) $$y_{c}=\frac{M_{01}}{M_{00}}=\frac{\sum_{x}\sum_{y}y\;\cdot f(x,y)}{\sum_{x}\sum_{y}f(x,y)}$$

The central position of the pupil can be determined using the geometric moments described above. According to Equations (2)–(4), the pupil size can be calculated directly from the zero-order moment, and the pupil center can be obtained by the ratio of the first-order moment to the area. The time complexity of locating the pupil center using geometric moments is O (*P*), where *P* is the number of nodes in the pupil area. This method exhibits low time complexity and excellent real-time performance.

This algorithm can handle different gray levels and pupil areas, demonstrating strong robustness to noise. The accuracy of the localization results is ensured through grayscale and geometric constraints. Since the algorithm does not require iterative operations or edge fitting, it minimizes the interference from noise points and improves the processing speed. Furthermore, the use of geometric moments for pupil center localization provides defocus adaptability, enabling reliable performance even in blurred images. In contrast, methods that rely on edge extraction and circle fitting are prone to errors in blurred images due to deviations in edge point selection, which have a significant impact on the accuracy of the pupil center localization. The proposed method calculates the geometric moments of the connected component, reducing the impact of edge contour diffusion in blurred images, thereby minimizing errors caused by deviations in edge point selection. Therefore, the proposed method enables the accurate localization of the pupil in blurred images.

## 3. Results

### 3.1. Experimental Dataset and Parameter Settings

The experimental data used during this study include the publicly available CASIA [23] dataset, the UTIRIS [24] dataset, the RONALDO [25] dataset, and a self-made dataset. The CASIA dataset has clear and high-quality images with a resolution of 320 × 280. The UTIRIS dataset has clear images but contains noise, which makes pupil localization more difficult, with a resolution of 1000 × 776. The RONALDO dataset, published in 2024, was developed using an eye-tracker and contains blurred human eye images with a resolution of 640 × 480.

In addition, in order to validate the practicality of the proposed algorithm in monitoring driver fatigue and in terms of its robustness to blurred images, we built a human eye image acquisition system and then acquired and produced a set of datasets containing out-of-focus images. The system’s focal length was set to 100 cm, with a defocus range of [−20, 20] cm and a resolution of 1920 × 1080. The image acquisition system consists of a camera and an 850 nm short-wave near-infrared (SW-NIR, 780 nm–1100 nm) LED light source. The 850 nm wavelength was chosen because common infrared sensors exhibit better responsiveness within this band. Additionally, infrared light at 850 nm operates at lower power levels, ensuring greater safety and posing no risk of harm to the human eye at the acquisition distance. The image acquisition experiments were conducted with the consent of the participants and under safe conditions.

### 3.2. Experiments and Results

The experiments were conducted on a computer with an Intel(R) Core(TM) i5-7400 CPU @ 3.00 GHz, 8 GB of RAM, running Windows 10. The algorithm was implemented using Visual Studio 2022 in combination with OpenCV 3.1.

#### 3.2.1. Experimental Results of the Proposed Algorithm

**Figure 9:**
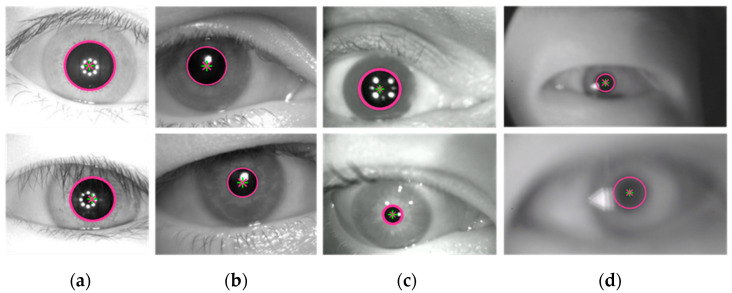
Localization results of the proposed algorithm. (**a**) CASIA dataset (clear); (**b**) UTIRIS dataset (clear); (**c**) RONALDO dataset (slightly blurred); (**d**) self-made dataset (strongly blurred). The red diagonal cross represents the ground truth of the pupil center, the green cross indicates the pupil center detected by the algorithm, and the red circle denotes the pupil contour identified by the algorithm.

To validate the effectiveness of the proposed algorithm, pupil localization experiments were conducted on the CASIA, UTIRIS, and RONALDO datasets, as well as a self-made dataset. The pupil localization results are shown in Figure 9. The experimental results demonstrate that the algorithm’s marked pupil center closely matches the ground truth, and the pupil boundary marked by the algorithm aligns well with the real pupil boundary. This confirms that the proposed algorithm can achieve accurate pupil localization in both clear and blurred eye images, exhibiting strong robustness and practicality.

**Figure 10:**
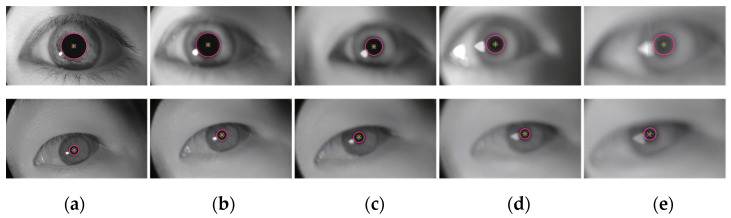
Pupil localization results of eye images with varying degrees of clarity. (**a**) *z* = 0 cm; (**b**) *z* = 5 cm; (**c**) *z* = 10 cm; (**d**) *z* = 15 cm; (**e**) *z* = 20 cm. The red diagonal cross represents the ground truth of the pupil center, the green cross indicates the pupil center detected by the algorithm, and the red circle denotes the pupil contour identified by the algorithm.

In order to further validate the defocus adaptability of the algorithm in the monitoring of fatigue driving, experiments were conducted using a self-made dataset containing blurred images. The pupil localization results of two randomly selected eye image sequences are shown in Figure 10. The blur level of the eye images captured by the eye-tracking device during actual driving is lower than that of images captured at a defocus of 20 cm [26]. The experimental results demonstrate that the proposed algorithm can achieve precise pupil localization under defocus conditions of less than 20 cm, thereby meeting the practical requirements for pupil localization in blurred images for driver fatigue monitoring.

To quantitatively evaluate the algorithm’s pupil localization performance at different levels of blur, this study collected 780 eye images at different defocus positions for pupil localization experiments. The localization accuracy was evaluated using two metrics: error and accuracy. Meanwhile, the algorithm’s runtime was recorded to assess its real-time performance. The error is defined as the Euclidean distance between the pupil center located by the algorithm (x,y) and the true value of the pupil center $\left(x_{0},y_{0}\right)$, measured in pixels. A smaller error indicates the higher localization accuracy of the algorithm, as expressed by the following formula:

(5) $$Error=\sqrt{{\left(x-x_{0}\right)}^{2}+{\left(y-y_{0}\right)}^{2}}$$

Due to slight annotation errors in the ground truth pupil centers, pupil localization is considered accurate when the localization error is within 1% of the number of pixels on the long side of the image. Accuracy is defined as the ratio of the number of correctly identified images to the total number of images. A higher accuracy indicates better localization performance by the algorithm. The expression is as follows:

(6) $$Accuracy=\frac{Number\;of\;correctly\;located\;samples}{Total\;number\;of\;samples}$$

Table 1 presents the pupil localization accuracy and runtime of the algorithm at different defocus positions. As shown in Table 1, when there is less than 20 cm of defocus, the localization error of the algorithm is within 6 pixels, the accuracy exceeds 97%, and the average localization time is 11.756 ms. The results indicate that the proposed algorithm meets the accuracy requirements for monitoring driver fatigue while demonstrating good real-time performance.

#### 3.2.2. Comparison of Algorithm Performance on Blurred Images

**Figure 11:**
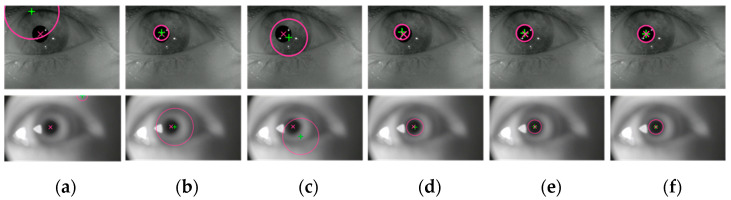
Pupil localization results of different algorithms. (**a**) GPIM; (**b**) HT; (**c**) FRST; (**d**) Ellseg; (**e**) PLNet; (**f**) ours. The red diagonal cross represents the ground truth of the pupil center, the green cross indicates the pupil center detected by the algorithm, and the red circle denotes the pupil contour identified by the algorithm.

To comprehensively evaluate the performance of the proposed algorithm, comparative experiments were conducted using three knowledge-based algorithms (GPIM [13], HT [14], and FRST [18]) and two data-driven methods (Ellseg [9] and PLNet [10]). GPIM, HT, and FRST were selected because they represent classic knowledge-based approaches. Ellseg and PLNet were chosen for their robustness in pupil localization, particularly in blurred images. These algorithms were applied to slightly blurred images from the RONALDO dataset as well as strongly blurred images from the self-made dataset. The pupil localization results of the different algorithms are shown in Figure 11.

As shown in Figure 11, the proposed algorithm demonstrates superior performance across human eye images with varying levels of blur. For slightly blurred images, GPIM and FRST fail to accurately localize the pupil center, while HT, Ellseg, PLNet, and the proposed algorithm achieve relatively accurate results. For images with higher blur levels, GPIM, FRST, and HT perform poorly, with Ellseg exhibiting some degree of error. However, both PLNet and the proposed algorithm maintain good accuracy. A quantitative evaluation of the performance of the algorithms is provided, and the results of the comparison are shown in Table 2.

As shown in Table 2, the proposed algorithm demonstrates significant advantages in both accuracy and real-time performance. Compared to classic knowledge-based algorithms, the proposed method incorporates grayscale and geometric constraints. This effectively reduces the impact of high-frequency information loss caused by image blur, resulting in higher localization accuracy. When compared to data-driven methods, the proposed algorithm improves localization accuracy by 18% over Ellseg, while performing similarly to PLNet. In addition, the proposed algorithm uses geometric moments to locate the center, eliminating the need for edge fitting, which improves the real-time performance. These enhancements allow the proposed algorithm to maintain high accuracy while meeting the real-time processing requirements.

#### 3.2.3. Comparison of Algorithm Performance on Clear Images

In order to objectively evaluate the performance of the proposed algorithm and ensure the fairness of the experiment, comparative experiments were conducted on two clear image datasets, CASIA and UTIRIS. The experimental results are shown in Table 3 and Table 4. Table 3 presents a comparison of the localization accuracy of the algorithms across the two datasets, while Table 4 shows a runtime comparison of the algorithms on the two datasets, with the unit in milliseconds (ms).

The experimental results show that all six algorithms exhibit good localization performance in the pupil center localization task for clear images. According to Table 3, HT, Ellseg, PLNet, and the proposed algorithm achieve the most accurate localization results, while the FRST algorithm shows minor errors, and the GPIM algorithm is sensitive to noise, resulting in instability. As shown in Table 4, GPIM and the proposed algorithm have the shortest runtimes, followed by PLNet, Ellseg, and FRST, with HT taking the longest time. Additionally, due to the larger image size and higher complexity of the UTIRIS dataset compared to the CASIA dataset, all algorithms perform better (in terms of accuracy and real-time performance) on the CASIA dataset than on the UTIRIS dataset.

By comparing the localization accuracy and runtime of the six algorithms, it can be observed that, for clear human eye images, the accuracy of the proposed algorithm is comparable to HT and data-driven methods such as Ellseg and PLNet, while significantly outperforming GPIM and FRST in terms of detection accuracy. Furthermore, our proposed algorithm reduces the computational time by 50% compared to HT and also shows some improvement over Ellseg and PLNet. These results demonstrate that, while maintaining high accuracy, the proposed algorithm offers a significant improvements in real-time performance, providing an efficient and accurate solution for pupil localization in monitoring driver fatigue.

## 4. Conclusions

In this study, we designed a real-time pupil localization algorithm for blurred images, based on double constraints, for driver fatigue monitoring. The algorithm first extracts the rough pupil area based on grayscale constraints and then refines the pupil area using a designed pupil shape index based on geometric constraints; finally, it locates the pupil center using geometric moments. By avoiding the need to fit a circle through feature points, the proposed algorithm mitigates the effects of edge diffusion in blurred images and reduces errors resulting from biased edge point selection. The algorithm demonstrates low time complexity, minimal parameter configuration requirements, and satisfactory real-time performance, making it suitable for applications in real-time driver fatigue monitoring. In future work, we plan to further investigate the performance of the pupil localization algorithm under various lighting conditions, such as visible light, to enhance its applicability. Additionally, the algorithm will be optimized for practical scenarios, including addressing challenges posed by drivers wearing glasses, prior to its deployment.

## Figures and Tables

**Table 1:** Pupil localization accuracy and time at different defocus positions.

Defocus Position (cm)	0	5	10	15	20
Average Error (pixel)	2.879	3.102	3.724	4.315	5.614
Accuracy (%)	100	99.4	99.4	98.1	97.4
Average Time (ms)	10.891	12.135	11.652	11.913	10.418

**Table 2:** Performance comparison of different algorithms.

	GPIM	HT	FRST	Ellseg	PLNet	Ours
Average error (pixels)	245.362	38.528	71.516	27.569	6.139	5.771
Accuracy (%)	32	64	58	83	96	98
Time (ms)	17.119	41.231	36.928	33.241	27.113	11.756

**Table 3:** Comparison of the localization accuracy of the algorithms.

Algorithm	CASIA		UTIRIS	
	Average Error (Pixels)	Accuracy (%)	Average Error (Pixels)	Accuracy (%)
GPIM	1.775	95.00	10.296	94.00
HT	1.275	97.75	4.711	96.25
FRST	1.300	96.25	5.663	95.50
Ellseg	1.241	97.75	4.615	97.00
PLNet	1.237	98.25	4.162	98.00
Ours	1.225	98.25	4.365	98.25

**Table 4:** Runtime comparison of the algorithms (unit: ms).

	GPIM	HT	FRST	Ellseg	PLNet	Ours
CASIA	12.326	27.915	21.556	19.124	18.203	7.025
UTIRIS	15.926	38.855	27.915	26.832	24.001	15.663

## Data Availability

Data are contained within the article.

## Abbreviations

The following abbreviations are used in this manuscript:

ROI	Region of Interest
GPIM	Gray Projecting Integral Method
FRST	Fast Radial Symmetry Transform
HT	Hough Transform
